# The effect of cathepsin K deficiency on airway development and TGF-β1 degradation

**DOI:** 10.1186/1465-9921-12-72

**Published:** 2011-05-31

**Authors:** Dongwei Zhang, Nelson Leung, Ekkehard Weber, Paul Saftig, Dieter Brömme

**Affiliations:** 1 The University of British Columbia, Department of Oral Biological and Medicinal Sciences, Vancouver, V6T1Z3, Canada; 2 Institute of Biochemistry, Martin-Luther University of Halle Wittenberg, Halle, Germany; 3 Unit of Molecular Cell Biology and Transgenic Research, Institute of Biochemistry, Christian Albrecht University Kiel, Germany

**Keywords:** lung airway, cathepsin K, TGF-β1, extracellular matrix, protease inhibitors

## Abstract

**Background**

Cathepsin K, a cysteine protease predominantly expressed in osteoclasts, is a major drug target for the treatment of osteoporosis. Recent findings, however, indicate that cathepsin K is also involved in non-skeletal metabolism. The development of fibrotic phenotypes in lung and skin is a concern for cathepsin K inhibitors presently evaluated in clinical trials. Cathepsin K is expressed in lung tissue and has been implicated in lung fibrosis. However, little is known about the role of cathepsin K in airway development and its effect on TGF-β1 degradation.

**Methods**

We investigated the effects of cathepsin K-deficiency on alterations in airway integrity, extracellular matrix composition, and TGF-β1 expression and degradation. Lung homogenates of wild-type and cathepsin K-deficient mice were used to evaluate their contents of collagen, glycosaminoglycans, and TGF-β1. The accessibility of TGF-β1 to cathepsin K-mediated degradation was determined *in vitro *and lung fibroblast proliferations in wild-type and cathepsin K-deficient cells were evaluated.

**Results**

Lung airway cathepsin K expression in wild-type mice remained constant between 1 and 6 months of age and the airway integrity was maintained. In contrast, after 2 months of age, all *Ctsk^-/- ^*mice demonstrated increased airway epithelium thickness by 16-28%, a lower structural airway integrity (1-2 score units lower), elevated cytokeratin expression of 12%, increased α-actin and vimentin expression by 50% and 70%, increased area of smooth muscle cells by 15%, elevated hydroxyproline and GAGs content by 20% and 25%, and increased TGF-β1 expression by 25%. TGF-β1 proved an efficient substrate of cathepsin K and TGF-β1 protein content in lung was increased by a potent cathepsin inhibitor. Lung fibroblasts from *Ctsk^-/- ^*mice after TGF-β1 treatment showed increased proliferation rates, increased levels of TGF-β1 by 30%, and increased ECM secretion.

**Conclusion**

This study suggests that airway development is partly regulated by cathepsin K and that its expression contributes to the maintenance of the airway structural integrity. The anticipated use of therapeutic cathepsin K inhibitors needs to take potential changes in human lungs into consideration.

## Background

Cathepsin K (CatK) is a lysosomal cysteine protease with potent collagenolytic and elastolytic activities. Its predominant expression in osteoclasts and synovial fibroblasts defined the protease as an important mediator of bone resorption and cartilage erosion [1-3]. Selective CatK inhibitors are presently being evaluated in clinical trials for osteoporosis [4,5]. However, at least one compound failed in phase II clinical trials due to severe skin side effects such as morphea [6]. One major concern of "off-site" effects of CatK inhibitors are fibrotic alterations in lung [7]. CatK downregulation is associated with the development of fibrosis in newborn lungs [8] and expression levels were significantly lower in lungs of premature infants developing bronchopulmonary dysplasia [9]. On the other hand, increased levels of CatK expression have been reported in lungs after bleomycin and silica treatment and correlated with fibrotic changes in the lung [10]. These data suggest that CatK plays a significant role in lung homeostasis.

Less is known about the effect of CatK activity on airway development and remodeling. Changes in airway structure are common to several pulmonary disorders, such as asthma and lung fibrosis. Changes are characterized by the reconstitution of the epithelium, airway smooth muscle cell hypertrophy and hyperplasia, abnormal deposition, and distribution of extracellular matrix (ECM) [11-13]. In airway remodeling, the equilibrium between production and degradation of ECM is disrupted, leading to a shift in the balance of synthesis and degradation of ECM and the abnormal deposition of matrix components. It has been reported that the secretion of growth factors such as TGF-β1 and altered expression of matrix degrading enzymes such as cathepsins [14] contribute to structural changes in the ECM. TGF-β1, one of the most potent regulators of connective tissue development, increases lung collagen deposition [15,16]. Elevated levels of TGF-β1 in lung fibroblast and epithelial cells are associated with the development of airway remodeling during asthma and correlate with the thickening of the basement membrane and the deposition of collagens [17].

Lung fibroblasts are important in generating a structural framework for the lung and also seem to be active participants in the remodeling process through proliferation and the production of specific mediators. It has been previously shown that epithelial cells, macrophages and fibroblasts express CatK in the lung [18,19] and that CatK protects against matrix deposition in bleomycin induced lung fibrosis [20]. Interestingly, the level of CatK expression in lungs of silica-treated mice was inversely related to the level of TGF-β1 expression suggesting a link between TGF-β1 and CatK [10].

The aim of this study is to investigate whether CatK expression directly contributes to the proper airway development via its ECM-degrading potential and/or indirectly by controlling TGF-β1 tissue contents.

## Methods

### Animals

*Ctsk^-/- ^*mice were generated as previously reported [21]. 10 female *Ctsk^-/- ^*and wild type (WT) mice (C57/BL6 from Jackson Lab) for each age group (1, 2, 3 and 6 months) were included in the study. Mice were fed a chow diet (Mouse Diet 5015, containing 11% fat). All animal experiments were performed in accordance with protocols approved by the local animal care advisory committee.

### Histological staining and quantitative image analysis

Mice were anesthetized and lungs were perfused with 10 mL ice-cold phosphate-buffered saline (PBS) through the right ventricle until lung cleared of blood. Then, the lungs were inflated with 10% formalin through the trachea, dissected and stored in 10% formalin for 24 h. Lungs were dehydrated and paraffin-embedded. Sections (5 μm) were processed for hematoxylin/eosin (H&E) and Masson trichrome staining. Microscopic images were acquired with the 20 × objective on a Leica microscope (Leica Microsystems Inc., Wetzlar, Germany) and evaluated with Openlab software (PerkinElmer, Waltham, Massachusetts, USA). The thickness of airway epithelium in H&E stained sections was measured from the base of the airway epithelium to the outer limit of the reticular lamina of the basement membrane at regular intervals of 20 μm with a digital micrometer as described [22]. The integrity of epithelium was assessed by a score system according to the percentage of length of the basement membrane with intact epithelium divided by the total length of the membrane. The intact epithelium has a single layer, orderly lining and no pseudo-stratified ciliated epithelium. The score system corresponds to the following grades: (1) 1, integrity < 15%; (2) 2, 15% ≤integrity < 40%; (3) 3, 40% ≤integrity < 65%; (4) 4, 65% ≤integrity < 85%; (5) 5, 85% ≤integrity. A 30 μm region extending from the airway basement membrane into the parenchyma was analyzed for collagen deposition by trichrome staining. The collagen content was quantified by the percentage of the area of collagen divided by the perimeter of the basement membrane. At least five distal airways from each mouse lung section (perimeter of basement membrane less than 950 μm) were selected for quantification.

### Hydroxyproline (HYP) assay and glycosaminoglycan (GAG) assays

Lung tissues were stored at -80°C after harvesting. The frozen lung was pulverized under liquid nitrogen in a mortar. The powdered tissue and the cell pellets were separately incubated with lysis buffer (150 mM NaCl, 10 mM Tris-HCl, pH 7.4, 1 mM EDTA, 1 mM EGTA, 1% Triton X-100, and 5 mM NaF) containing a protease inhibitor cocktail (Roche, Mannheim, Germany), and were further homogenized by ultrasonication. The supernatants and pellets were separated by spinning at 14,000 g for 20 min at 4°C and protein concentrations were determined using the Bradford assay. The supernatants were kept for immunoblot analysis. The protein from cell culture media was precipitated by addition of 10% trichloroacetic acid in ice cold acetone. Homogenized lung tissue and the precipitations from the media were dried under vacuum at 60°C until a constant weight was obtained. The dried homogenized lung tissue and precipitations were hydrolyzed by autoclaving at 120°C for 20 min twice in the presence of 2 N NaOH. The hydrolysate was used for the HYP assay according to Reddy et al. [23]. After incubation, triplicates of 200 μl of supernatant of the reaction solution were transferred to a 96-well plate and the absorbance at 570 nm was measured within 30 min. The results were calculated as μg of HYP per mg dry tissue weight. Total sulfated glycan content was measured using Blyscan Sulfated Glycosaminoglycan Assay kits (Biocolor, Carrickfergus, County Antrim, UK) according to the manufacturer's instructions. All data were sampled in triplicates.

### Immunohistochemical staining

Sections were incubated with 1% bovine serum albumin for 30 min at room temperature and subsequently incubated at 4°C overnight with mouse anti cathepsin K (1H6: 1:100 dilution, produced by Dr. E. Weber), mouse anti α-smooth muscle actin (1:400, Sigma, Saint Louis, MO) and goat anti-vimentin (1:150, Sigma). Sections were then rinsed and incubated with FITC-conjugated goat anti mouse IgG (1:100, Rockland Immunochemicals Inc., Gilbertsville, PA) and rabbit anti goat IgG (1:100, Rockland) for 30 min at room temperature. For controls, the primary antibodies were replaced by non-immunized goat serum. In the case for CatK immunostaining, *Ctsk^-/- ^*lung tissue was used. Morphometric quantification of CatK expression in WT mice airways was performed by measuring the area of CatK positive staining inside the airway basement membrane normalized to the perimeter of the basement membrane. Morphometric quantification of airways was performed by calculating the area of actin and vimentin staining encircling the airway divided by the perimeter of the basement membrane. The size of airway SMCs within the sampling area was measured by the total area of airway encircling SMCs (actin staining) divided by the numbers of airway SMCs. For each mouse, five airways were analyzed.

### Immunoblot analysis

Proteins in lung homogenates and cell lysates were separated by SDS-PAGE and were then transferred onto PVDF membranes. Membranes were incubated with the appropriate primary antibody overnight at 4°C. The concentrations of primary antibodies were as follows: monoclonal mouse anti-α smooth muscle actin (1:5000, Sigma), β-actin (1:10000, Sigma), polyclonal goat anti-vimentin (1:500), rabbit anti-cytokeratin (1:1000, Sigma) and chicken anti-TGF-β1 (1:850, R&D, Minneapolis, MN). Blots were then washed with PBS Tween-20 (PBST) for 10 min three times and incubated for 1 h at room temperature with horseradish peroxidase-conjugated anti-mouse (1:3000, Southern Biotech, Birmingham, AL), anti-goat (1:3000, Southern Biotech), anti-rabbit (1:3000, Southern Biotech) and anti-chicken antibodies (1:1500, R&D), respectively, diluted in PBST. Blots were rinsed 3-times with PBST for 10 min each at room temperature. Then images of the blots were captured and analyzed using the Chemigenius imaging system (Syngene, Cambridge, UK). All values were normalized relative to β-actin loading control. Pixel densities were corrected for background staining in the same membrane.

### Cathepsin-dependent TGF-β1 degradation

The pH value of lung homogenates from *Ctsk^-/- ^*mice were adjusted to pH 5.5 by 3 M acetate buffer. Then 2.5 mM dithiothreitol (final concentration) was added to lung homogenates. The TGF-β1 expression was investigated by two different assays. First, the lung homogenates were incubated with or without human recombinant CatK (1.15 nM, final concentration) at room temperature. Human CatK was expressed in *Pichia pastoris *as previously described [24]. At 0, 0.5 h, 1 h, 2 h, and 4 h, lung homogenates were transferred into a new tube containing 10 μM E-64 (final concentration) and were incubated another 10 min at room temperature. Second, the lung homogenates were incubated with or without LHVS (10 μM, final concentration) at 650 rpm 28°C for 8 hours. The final products of both assays were analyzed by immunoblot using chicken anti-TGF-β1 (R&D) as primary antibody and corresponding horseradish peroxidase-conjugated rabbit anti-chicken IgY (R&D) as secondary antibody.

### Primary mouse lung fibroblasts (MLFs) culture and treatment

After anesthesia, the thorax was opened and lungs were perfused with sterile PBS via the right ventricle until they were pale. Tissues were minced and suspended in Dulbecco's modified Eagle's medium (DMEM, Invitrogen, Carlsbad, CA) containing 0.2% collagenase IV and 0.01% DNase I and incubated with gentle stirring at 37°C for 30 min. After dispersion with collagenase, isolated cells were centrifuged, washed, and cultured in DMEM supplemented with 10% fetal bovine serum (FBS), 1% of L-glutamine, and penicillin, streptomycin, and fungizone. Cultures were re-fed every other day. All cultures were evaluated by immunohistochemistry to assess the expressions of vimentin and cytokeratin. All stained positively with vimentin and but failed to stain for the epithelial cell marker, cytokeratin. Fibroblasts between 2 and 3 passages were used for the assays.

Fibroblasts were incubated in 150 mm plates in 10% FBS/DMEM to reach 70% confluence prior to treatment. TGF-β1 was reapplied every 24 h. At indicated time points, the media was removed from the cells and cells were washed twice with ice-cold PBS. In some experiments, at various time points after incubation of cells, the overlying cell-conditioned medium was removed, stored in sterile tubes in the presence of a protease inhibitor cocktail (Roche, Mannheim, Germany) and frozen at -80°C until used.

### Crystal Violet, MTT and wound healing assays

The Crystal Violet assay (CVA) was used to determine fibroblast proliferation. Primary lung fibroblasts were trypsinized and sub-cultured in 24-well plates. Cells were grown in 10% FBS in DMEM to reach 70% confluence, and were then starved with serum free DMEM for 24 h. After starvation, FBS concentration was decreased to 2.5% in the presence or absence of TGF-β1. At selected time points, the cell layer was washed once with PBS. Cells were then fixed and stained by 0.5% crystal violet solution in 25% methanol. After 10 min, the excess dye was removed by washing with tap water. Then, the incorporated dye was solubilized in 1% SDS in PBS, and 200 μl were transferred to a 96-well plate. To determine the cell numbers in each sample, the optical density (OD) was measured directly at a wavelength of 570 nm using a Spectramax Plus reader (Molecular Devices, Sunnyvale, CA).

For the verification of the CVA, the MTT assay [thiazolyl blue, 3-(4,5-dimethylthiazol-2-yl)-2,5-diphenyltertrazolium bromide] (Sigma) was performed to determine fibroblast cell viability and proliferation. On specified time points, the culture media was removed, and fibroblasts were incubated with 0.5 mg/ml MTT solution in an incubator at 37°C for 3 h. After removing the medium, 600 μl of DMSO was added to each well of a 24-well plate to solubilize the blue-colored tetrazolium and the plates were then shaken for 5 min. 200 μl of MTT solution were transferred to a 96-well plate. The absorbance at 570 nm was monitored using a Spectramax Plus reader. Data were expressed as percentage of OD_570 nm _of TGF-β1 treated sample compared to that of TGF-β1 untreated samples.

Cell scratch assays were used to determine the migration and proliferation rate of cells. Equal numbers of fibroblasts were seeded onto 12-well plate. After 90% confluence being reached, cells were rinsed with PBS and starved in serum free medium for 24 h. Then three uniform wounds were created per well by gently scratching a sterile P-200 pipette tip across the surface of the cultures. Cultures were washed with PBS to remove cellular debris, and cultures were allowed to subsequently incubated for 24 h in DMEM supplemented with 10% FBS. Phase-contrast photomicrographs of the wounds were obtained at 0 and 24 h after injury. The wound area was quantified using the Openlab software. The percentage of the wound healed was determined by the healing area (0 h wound area subtracted by 24 h wound area) divided by the 0 h wound area.

### TGF-β1 protein determination by ELISA

Total TGF-β1 content in cell culture supernatants and lung homogenates were determined by ELISA. This assay was performed according to the manufacturer's recommendations (Invitrogen). TGF-β1 concentration was normalized to total protein as determined by micro Bradford Protein Assay. Data are expressed as pg TGF-β1 per mg of protein.

### Statistical analysis

Results are expressed as mean ± SE. All image analyses were performed by two observers blinded to the group status. Score comparisons between groups were performed using the Chi-test. The significance of differences of the mean values was calculated using the one-way ANOVA (t-test). A p value of less than 0.05 was considered significant.

## Results

### The expression of CatK in mouse small airways

**Figure 1:**
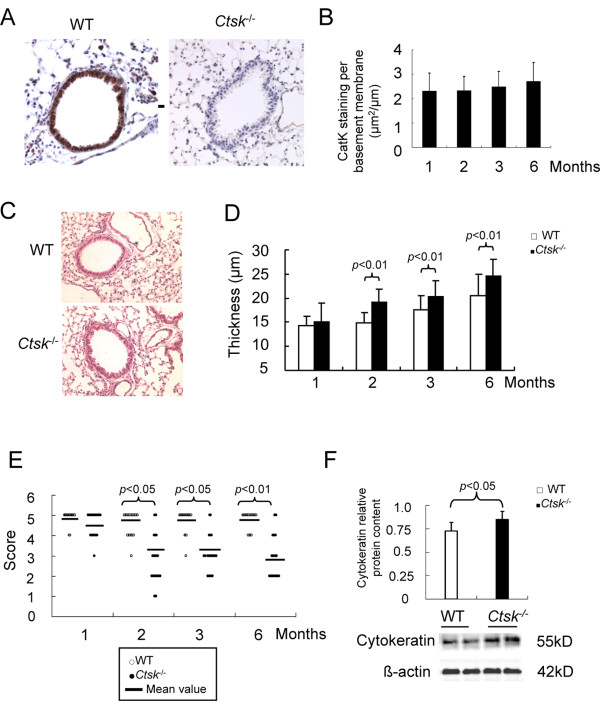
**Expression of CatK in mouse lungs and its effects on airway epithelium**. Representive immunohistochemical staining (A) of a small airway from a 3 months old WT mouse and an age matched *Ctsk^-/- ^*lung tissue sample (original magnification, ×200). (B) Morphometric analysis of CatK expression in airway epithelium from mice of various ages. The density of CatK expression was normalized to the perimeter of the basement membrane. Representative H&E staining (C) of small airways from 2 months old WT and *Ctsk^-/- ^*mice (original magnification, ×200). Morphometric analysis shows epithelium thickness (D) and integrity (E) in lung sections from mice of various ages. Representative image and band analysis (F) of immunoblots show cytokeratin expression in 2 month old lung homogenates. The results revealed that airway epithelium was increased in thickness and diminished in integrity in *Ctsk^-/- ^*mice. Morphometry and image analysis were processed using Openlab software and Chemigenius system, respectively. All values are expressed as means ± SE for 5 separate experiments. (●(WT) and ◆(*Ctsk^-/-^*) indicate the number of mouse at a certain score.

Using a monoclonal cathepsin K antibody (1H6) we observed staining for Catk in airway epithelial cells, in pneumocytes, and likely in infiltrated macrophages (Figure 1A left panel). *Ctsk^-/- ^*derived tissue did not reveal staining indicating the specificity of the antibody used (Figure 1A, right panel). The total airway epithelial area positive for CatK staining per μm of airway perimeter did show a trend of increased CatK expression from one to six months (approximately 17% increase in mean values) but there was no significant increases between the individual time points (1, 2, 3, 6 months of age; Figure 1B), indicating a relatively constant CatK expression in the age groups evaluated.

### Effect of CatK deficiency on lung development

The airway epithelium of WT mice is organized in a single layer displaying an orderly lining (Figure 1C). In contrast, the airway epithelium of *Ctsk^-/- ^*mice of two months and older is significantly thicker and disorganized (Figure 1C). From month 2 to 6, the epithelium was about 16-28% thicker in *Ctsk^-/- ^*mice when compared to WT littermates (Figure 1D). The integrity of the airway epithelium is also altered. In some cases, large zones of epithelium were damaged and disrupted. Using an epithelium integrity score, we observed that from 2 months on, *Ctsk^-/- ^*mice exhibited integrity scores 1-2 units below that of WT littermates (p < 0.05) (Figure 1E). These alterations were also confirmed by immunoblot analysis evaluating the expression of cytokeratin as a marker of epithelial cells. The expression of cytokeratin was approximately 12% higher in *Ctsk^-/- ^*mice and correlated with the thickness differences between the two mouse strains (Figure 1F).

**Figure 2:**
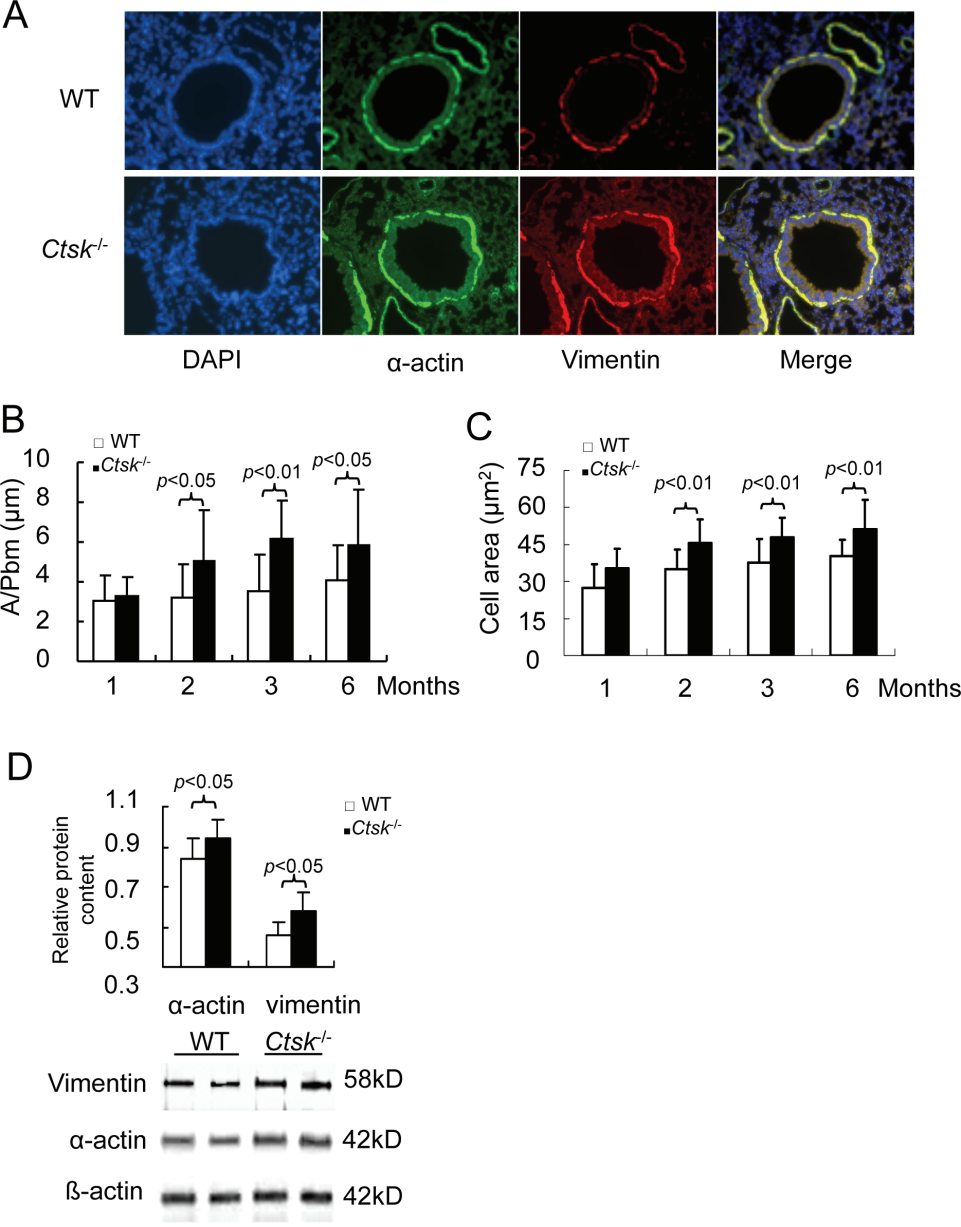
**Effects of CatK expression on the airway smooth muscle cells**. (A) Representative immunofluorescence staining of small airways from 2 months old WT and *Ctsk^-/- ^*mice (original magnification, ×200). Smooth muscle cell layer thickness and cell area in lung sections from mice of various ages are depicted in panels B and C. (D) Representative image and band intensity analysis data of immunoblots show increased expression in α-actin and vimentin in 2 months old WT and *Ctsk^-/- ^*mice. Morphometry and image analysis were processed using Openlab software and Chemigenius system, respectively. A/Pbm means the staining area divided by the perimeter of airway basement.

α-actin and vimentin, which are important for cell contraction and motility, are marker proteins for assessing the phenotype of SMCs and myofibroblasts [25]. Immunofluorescence analysis showed that the staining for α-actin and vimentin completely overlapped indicating that the subepithelial cell layer represents a uniform cell type (Figure 2A). These cells revealed that the α-actin/vimetin positive area per micrometer of internal airway perimeter increased by 50 to 70% (Figure 2B) reflecting both cell size and cell number increases. The average size of SMCs was increased by 14-16% in *Ctsk^-/- ^*mice from 2 months to 6 months of age (Figure 2C). All changes were significant from the age of 2 months (p < 0.05). These changes were also corroborated by immunoblot analysis of lung homogenates of 2 month old mice. The expression of α-actin and vimentin was elevated by 12% and 27% in *Ctsk^-/- ^*mice compared to WT littermates (Figure 2D).

### Effect of CatK deficiency on extracellular matrix content

**Figure 3:**
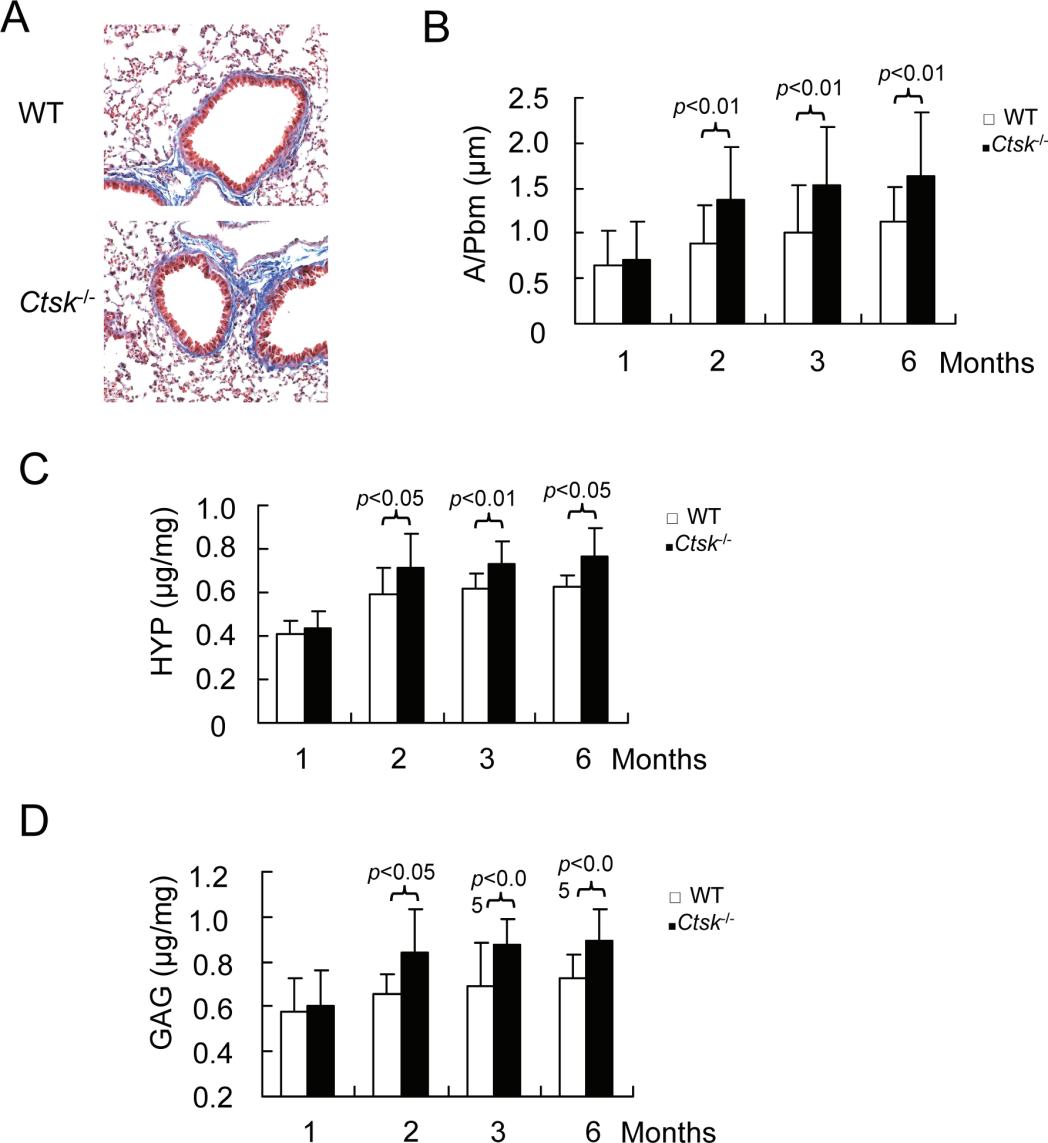
**Effects of CatK deficiency on ECM content in lung**. (A) Representative Masson trichome staining of small airways from 3 months old WT and *Ctsk^-/- ^*^- ^mice shows collagen distribution around airways (blue color; original magnification, ×200). (B) Morphometric analysis of collagen distribution surrounding the airways. (C) hydroxyproline (HYP) and (D) glycosaminoglycan (GAG) contents in lung sections from 1, 2, 3, 6 months old mice. The unit (μg/mg) indicates the HYP and GAG contents per mg protein. Morphometric analyses were performed using Openlab software. A/Pbm means the staining area divided by the perimeter of airway basement.

The sub-epithelial layer is composed of ECM components such as collagen and GAGs. Changes in lung ECM content may affect the contractile properties and the phenotype of airway SMCs [26]. To analyze the ECM content, we first used Masson's trichrome staining (Figure 3A) to evaluate the collagen content surrounding the airways. *Ctsk^-/- ^*mice displayed a significant increase in collagen deposition which extended through the epithelial barrier and included the perivasculature, when compared to same age WT littermates (about 45% increase from 2 months of age onwards) (Figure 3B). Secondly, we determined the collagen (HYP) and GAG contents of whole lung tissue extracts which revealed significant increases by 20% and 25%, respectively, in *Ctsk^-/- ^*mice when compared to WT littermates from 2 months of age onward (p < 0.05) (Figure 3C, D). These results suggest a role of CatK in the homeostasis of the ECM surrounding the airways.

### Effect of CatK deficiency on TGF-β1contents in lung

**Figure 4:**
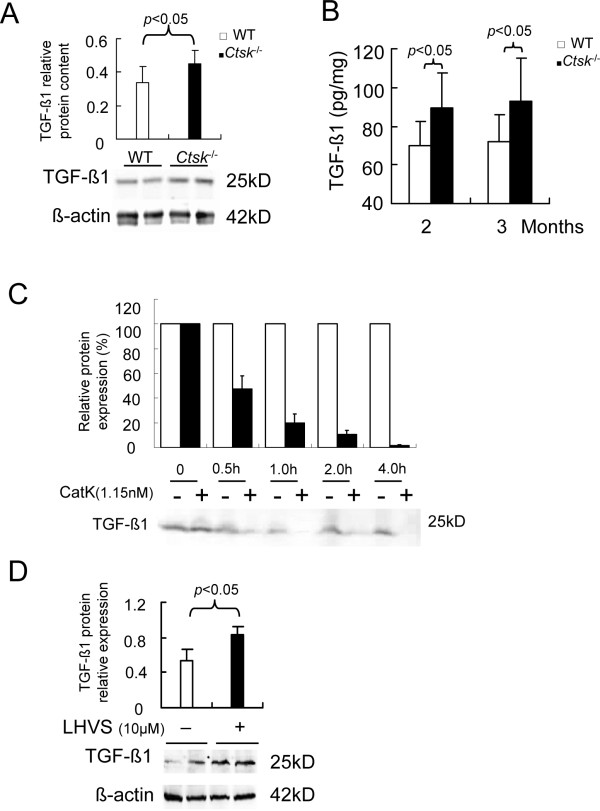
**Effects of CatK expression on TGF-β1 tissue content and secretion**. Representative image and band analyses (A) of immunoblots from 2 months old WT and *Ctsk^-/- ^*mice lung homogenates show upregulation of TGF-β1 expression in *Ctsk^-/- ^*mice lung. (B) ELISA results show TGF-β1 secretion is increased in the *Ctsk^-/- ^*^- ^mice lung. (C) Relative amount of TGF-β1 in lung homogenate in the presence or absence of recombinant human CatK (1.15 nM). (D) shows TGF-β1 expression in the absence or presence of the cathepsin inhibitor LHVS. Image analysis was performed using Openlab software and Chemigenius system, respectively. All immunoblot experiments were performed in triplicates. The relative protein expression relates to the expression of β-actin.

To investigate whether CatK expression-associated changes in airway development depend on a CatK-dependent regulation of TGF-β1, the levels of TGF-β1 in lung homogenates of 2 and 3 months old mice were determined by immunoblot and ELISA. Both assays revealed that the expression of TGF-β1 was significantly higher (about 25%) in *Ctsk^-/- ^*mice than in the WT littermates (p < 0.05, Figure 4A, B). To further elucidate the interaction between CatK and TGF-β1, we studied the *in vitro *digest of lung tissue TGF-β1 by recombinant CatK. Results depicted in Figure 4C show a 50% degradation of TGF-β1 after 30 min and a complete degradation after 4 h. The *in vitro *cleavage specificity of CatK towards TGF-β1 is corroborated by the finding that α-actin was insignificantly degraded by CatK in the same assay (data not shown). We also studied TGF-β1 expression in presence of LHVS (a pan-cathepsin vinyl sulfone inhibitor at 10 μM concentration [27]). Results depicted in Figure 4D show that the TGF-β1 content in lung tissue was markedly increased by 53% in the presence of LHVS. Taken together, these results suggest that CatK can significantly influence the TGF-β1 content in lungs.

### Effect of CatK-deficiency on mouse lung fibroblast-like cells (MLFs)

**Figure 5:**
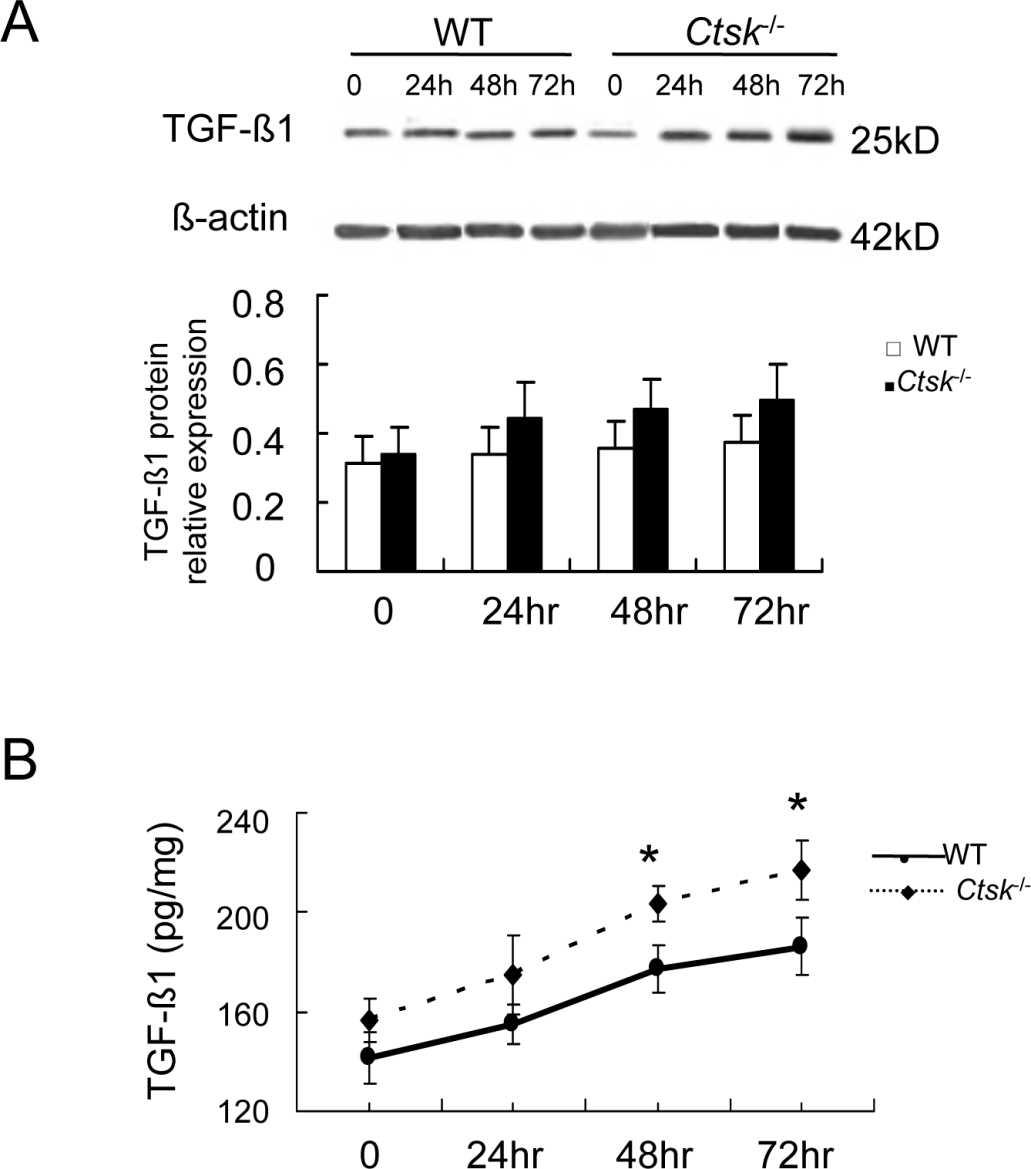
**Effect of CatK expression on TGF-β1 expression and secretion in mouse lung fibroblast (MLFs)**. (A) shows representative images of immunoblot and band analysis of TGF-β1 expression in WT and CatK-deficient MLFs at indicated time points. (B) shows ELISA analysis of TGF-β1 secretion from MLFs into conditioned media. (* p < 0.05, ** p < 0.01 compared with same time point. All experiments were performed three or four times.)

The evaluation of α-actin/vimetin-positive cells surrounding small airways revealed an increase in their numbers and sizes in *Ctsk^-/- ^*mice when compared to WT littermates (Figure 2B, C). Therefore, we studied the effect of CatK-deficiency on these cells *in vitro *regarding the expression/content of TGF-β1 and ECM components. MLFs were treated with 5 ng/ml recombinant human TGF-β1 at the indicated time points and then TGF-β1 levels were measured by immunoblot analysis and ELISA. As shown in Figure 5A, the expression of TGF-β1 protein in *Ctsk^-/- ^*MLFs was increased to 130% at 24 hours (p < 0.05) when compared with WT MLFs and remained at this level until 72 hours (p < 0.01). In conditioned cell culture media, ELISA (Figure 5B) results revealed similar increases in TGF-β1 in *Ctsk^-/- ^*mice.

**Figure 6:**
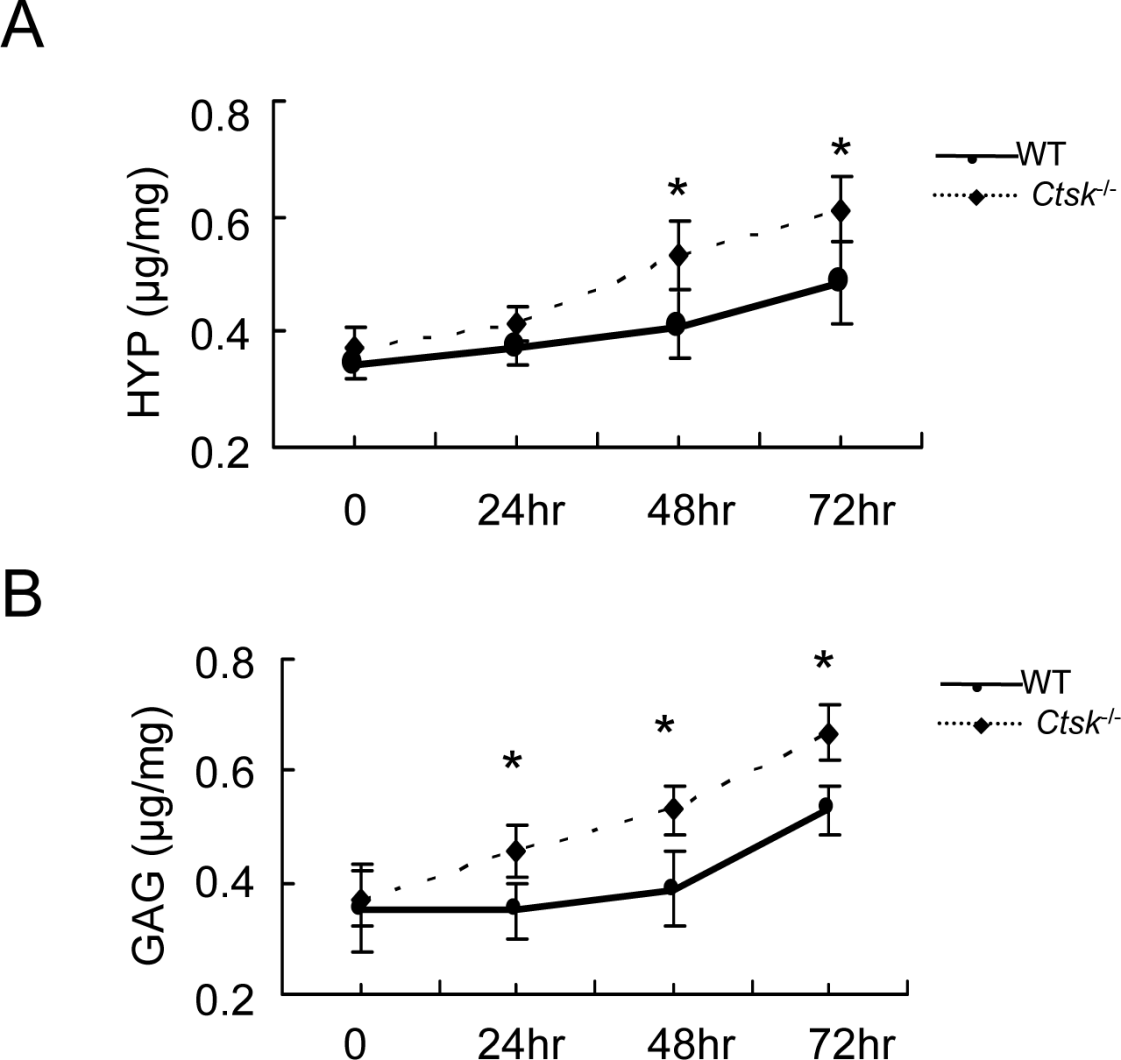
**HYP and GAG levels in conditioned media of mouse lung fibroblast cultures from WT and CatK-deficient mice (MLFs)**. (A) HYP content; (B) GAG content. The unit (μg/mg) indicates HYP or GAG content per mg protein in the media. * p < 0.05, ** p < 0.01 compared with same time point.

TGF-β1 can induce fibroblasts to undergo differentiation into a myofibroblast phenotype [15]. The differentiated myofibroblast has an increased capacity to express contractile proteins and to deposit collagen [28,29]. Accordingly, we investigated whether alterations of ECM expression in lung fibroblasts depends on the expression of CatK. We studied the effect of CatK on changes of ECM production in MLFs using the HYP and GAG assays. In the conditioned media, the HYP content, a marker for collagen, increased after 24 h by 12% and stayed at 130% after 48 and 72 h in *Ctsk^-/- ^*MLFs whereas the GAG content was 12-14% higher in *Ctsk^-/- ^*MLFs than in WT MLFs (Figure 6A, B). The increases in HYP and GAG in the culture media must therefore reflect an increase in the expression and/or secretion of matrix components by *Ctsk^-/- ^*MLFs.

### Effect of CatK deficiency on TGF-β1 stimulated proliferation of mouse lung fibroblast (MLF) and wound healing

**Figure 7:**
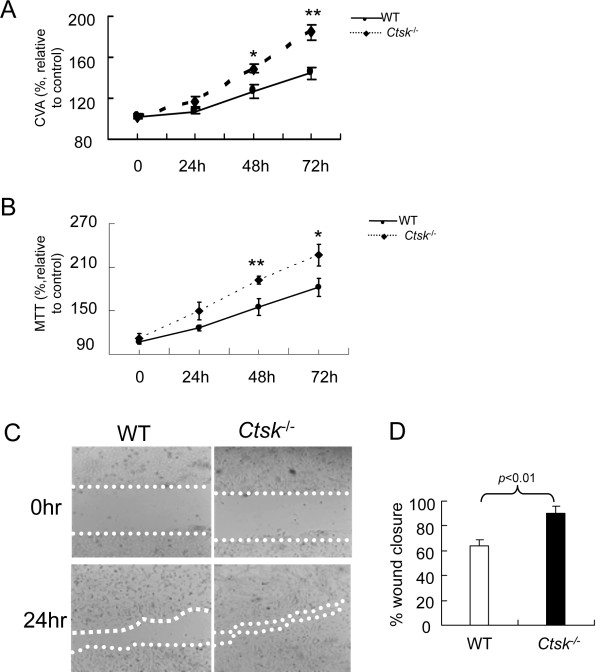
**Effects of CatK expression on the migration and proliferation of MLFs**. Quantitative analysis of crystal violet assay (CVA) (A) and MTT assay (B) after MLFs were incubated with TGF-β1 (5 ng/ml) for 0-72 h. (C) phase images of wound healing and wound closure rates (D) show that *CatK-deficient *MLFs facilitate wound closure. * p < 0.05, ** p < 0.01 compared with same time point.

To evaluate the role of CatK expression on TGF-β1 mediated cell proliferation of MLFs, we analyzed their proliferation rates using three different assays: i) CVA, ii) MTT assay, and iii) cell scratch assay. Cell growth rates were monitored at 0, 24, 48, and 72 h. The addition of TGF-β1 increased both WT and *Ctsk^-/- ^*fibroblasts cell growth rates when compared to the controls. However, between 24 and 72 h, the proliferative response of *Ctsk^-/- ^*fibroblasts to TGF-β1 was between 20 and 40% higher than that in WT fibroblasts (p < 0.05, Figure 7A, B). These results suggest that CatK expression can attenuate the cell proliferative effect of TGF-β1 in MLFs. As expected from the proliferation data, the wound healing rate for *Ctsk^-/- ^*MLFs was about 40% higher than that of WT cells (Figure 7C). These findings indicated that *Ctsk^-/- ^*MLFs migrated and proliferated much faster than WT MLFs.

## Discussion

Airway remodeling is observed in various lung diseases such as asthma and chronic obstructive pulmonary disease [30]. Besides changes in the cellular composition of airways, airway remodeling is characterized by an imbalance between excessive extracellular matrix production and their proteolytic turnover. Proteolytic activities such as matrix metallo- and cysteine proteases have been implicated in lung tissue remodeling [31,32]. More recently, attention was given to CatK, a papain-like cysteine protease with a potent collagenase and elastase activity. Excess as well as deficiency in CatK expression have been linked to pathological phenotypes. CatK-deficient mice in the bleomycin-induced lung fibrosis model display a stronger fibrotic phenotype than their WT littermates. The phenotype is characterized by a build-up of collagen fibers due to the lack of the collagenase activity [33]. On the other hand, overexpression of CatK leads to increased collagen degradation and a partial protection from bleomycin and silica-induced fibrosis [10,20]. It was shown that TGF-β1 down-regulates the expression of CatK in silica-induced lung fibrosis and that the level of CatK expression is inversely related to the expression of TGF-β1 and the susceptibility to lung fibrosis [10]. In our present study, we specifically focused on the effect of CatK deficiency on airway development and suggest that the role of CatK is not limited to its matrix protein degrading activity but also affects the proteolytic control of the profibrotic factor, TGF-β1. Our results demonstrated that CatK deficiency in mice correlates with increased thickness and reduced structural integrity of the epithelium, an increase in the sizes and numbers of SMCs as well as with elevated contents of collagen and GAGs. These changes appear to be in part induced by increased levels of TGF-β1 in *Ctsk^-/- ^*lungs. CatK can reduce the tissue content of TGF-β1 by its ability to specifically degrade the growth factor. Furthermore, lung fibroblasts from *Ctsk^-/- ^*mice showed increased migration and proliferation rates. TGF-β1 expression and ECM production were more evident in *Ctsk^-/- ^*lung fibroblasts than in WT lung fibroblasts. Interestingly, the specific CatK inhibitor, balicatib, was reported to increase intact parathyroid hormone levels in clinical human trials for osteoporosis by 50% (discussed in [34]) corroborating the potential of CatK to degrade regulatory factors.

The increased thickness of the epithelium and its loss of integrity in *Ctsk^-/- ^*mice can be understood in several ways. First, the proliferation of airway epithelium dominates over apoptosis and necrosis [35]. The proliferative response in the airway epithelium may result in epithelial hyperplasia and thickening resulting in a disorganized epithelium. Cathepsin expression has been implicated in apoptosis [36] and we have evidence that overexpression of CatK increases apoptosis rates in lung fibroblasts (Zhang and Bromme, unpublished data). Second, the proliferative underlying cells such as SMCs and fibroblasts may migrate into the airway epithelium. Under the stimuli from mediators such as TGF-β1 these migrating cells may be transformed into myofibroblasts [15] which will produce more matrix proteins [37]. Third, the predominance in mesenchymal cells (including fibroblasts, myofibroblasts, and SMCs) and the lack of CatK expression may also increase the deposition of ECM under the basement membrane [38]. The abnormal ECM surrounding the airways may secret more mediators, which promotes epithelial thickening and remodeling [39]. The ECM itself may also affect the growth of airway epithelial cells and influence epithelial thickness [40]. Taken together, all these changes favor alterations in the airway structure.

Hypertrophy and hyperplasia of SMCs within the airway contribute to changes in the airway structure [41,42]. This study is the first to report that SMC hypertrophy is linked to CatK deficiency. The propensity for SMCs to grow when CatK is deficient may be linked to the sensitivity of the SMC to growth factors and altered pattern of matrix composition [43]. We observed epithelium thickening and loss of structural integrity as well as abnormal subepithelial collagen deposition within the airway wall. We also revealed that the level of TGF-β1 protein content in whole lung homogenates has been elevated in *Ctsk^-/- ^*mice. This was consistent with van den Brûle's results that the level of CatK expression in silica-induced lung fibrosis was inversely related to the level of TGF-β1 expression [10]. The elevated level of TGF-β1 may induce SMC hypertrophy and hyperplasia [44]. On the other hand, the increasing ECM deposition in *Ctsk^-/- ^*mice may promote the survival, proliferation, migration and cytokine synthesis of airway SMCs [45]. TGF-β1 may induce fibroblast differentiation into myofibroblasts [46] which results in increased ECM production. Then the ECM may subsequently act as a proliferation and migration factor for fibroblasts, thus leading to further airway remodeling. In this study, we have demonstrated that lung homogenates from *Ctsk^-/- ^*mice released more TGF-β1, which in turn led to an increase in ECM components such as HYP and GAGs. The TGF-β1 content in lung homogenates could be also increased by the addition of a potent CatK inhibitor such as LHVS. The increased TGF-β1 level was also accompanied by an increased thickness of the surrounding airway smooth muscle layer.

There is evidence that fibroblasts are the main cellular source of the extracellular collagen matrix deposition [47,48]. These cells are themselves excellent sources of CatK [18,33] and TGF-β1 [49]. Under physiological condition, fibroblasts responding to mediators (including TGF-β1 produced by parenchymal cells) can release TGF-β1. Our data indicate that CatK can decrease TGF-β1 tissue levels and therefore control its overall expression and subsequent secretion. It is tempting to speculate that CatK acts as a regulator of TGF-β1 contents produced by fibroblasts. In *Ctsk^-/- ^*mice, TGF-β1 levels were elevated. Increased concentrations of TGF-β1 could then drive airway remodeling both through cell proliferation and enhanced matrix production. Although our data clearly suggest that CatK is involved in TGF-β1 regulation, many questions such as the exact site of TGF-β1 degradation (extra- or/and intracellular) as well as the potential proteolytic effect of CatK on the TGF-receptor remain open.

In this study, we demonstrated that CatK deficiency mediates airway development and reason that TGF-β1 signaling is partly controlled by the degradation of the growth factor by CatK. The hypothesis that CatK can increase TGF-β1 tissue levels is supported by several lines of evidence. First, we have shown that TGF-β1 levels are elevated in lungs of *Ctsk^-/- ^*mice and CatK-deficient MLFs when compared to the growth factor levels in WT mice and MLFs. Second, we demonstrated that CatK can specifically decrease TGF-β1 levels in *Ctsk^-/- ^*lung homogenates and that TGF-β1 expression is increased in the presence of the cathepsin inhibitor, LHVS. Third, it has been shown by others that the level of CatK transcription is strongly upregulated following silica treatment, and that CatK expression was negatively correlated with TGF-β1 expression [10]. Altogether, these results suggested that interactions between CatK and TGF-β1 contribute to airway development.

In conclusion, we demonstrated that CatK plays a significant role in mouse airways. Our findings caution that anti-CatK targeted therapies such as presently developed for the treatment of osteoporosis may have the potential to induce lung remodeling processes. Further elucidation of the role of CatK in healthy and diseased airways will provide us with invaluable insight into the processes that lead to the development of lung diseases.

## Declaration of competing interests

The authors declare that they have no competing interests.

## Authors' contributions

DWZ designed and carried out experiments, analyzed data and wrote the paper, NL carried out MTT and CV assays, participated in histological scoring of airway integrity, EW, provided the mab for cathepsin K, PS provided the Ctsk^-/- ^mice, DB conceived experiments, analyzed data and wrote the paper. All authors have read and approved the final manuscript.
